# Correction: FOXP4 Is a Direct YAP1 Target That Promotes Gastric Cancer Stemness and Drives Metastasis

**DOI:** 10.1158/0008-5472.CAN-25-5025

**Published:** 2026-01-16

**Authors:** Xiaoli Liu, Bonan Chen, Fuda Xie, Kit Yee Wong, Alvin H.K. Cheung, Jinglin Zhang, Qian Wu, Canbin Fang, Jintao Hu, Shouyu Wang, Dazhi Xu, Jianwu Chen, Yuzhi Wang, Chi Chun Wong, Huarong Chen, William K.K. Wu, Jun Yu, Michael W.Y. Chan, Chi Man Tsang, Kwok Wai Lo, Gary M.K. Tse, Ka-Fai To, Wei Kang

In the original version of this article (1), the Materials and Methods was missing a section describing the *Yap1*/*Taz* double-knockout mouse model. Additionally, the supplemental figure legends were inadvertently omitted. The errors have been corrected in the latest online HTML and PDF versions of the article. The authors regret these errors.
